# Retroviral DNA Sequences as a Means for Determining Ancient Diets

**DOI:** 10.1371/journal.pone.0144951

**Published:** 2015-12-14

**Authors:** Jessica I. Rivera-Perez, Raul J. Cano, Yvonne Narganes-Storde, Luis Chanlatte-Baik, Gary A. Toranzos

**Affiliations:** 1 Department of Biology, University of Puerto Rico, Rio Piedras Campus, San Juan, Puerto Rico; 2 Center for Applications in Biotechnology, Biological Sciences Department, California Polytechnic State University, San Luis Obispo, California, United States of America; 3 Center for Archaeological Research, University of Puerto Rico, Rio Piedras Campus, San Juan, Puerto Rico; University of Florence, ITALY

## Abstract

For ages, specialists from varying fields have studied the diets of the primeval inhabitants of our planet, detecting diet remains in archaeological specimens using a range of morphological and biochemical methods. As of recent, metagenomic ancient DNA studies have allowed for the comparison of the fecal and gut microbiomes associated to archaeological specimens from various regions of the world; however the complex dynamics represented in those microbial communities still remain unclear. Theoretically, similar to eukaryote DNA the presence of genes from key microbes or enzymes, as well as the presence of DNA from viruses specific to key organisms, may suggest the ingestion of specific diet components. In this study we demonstrate that ancient virus DNA obtained from coprolites also provides information reconstructing the host’s diet, as inferred from sequences obtained from pre-Columbian coprolites. This depicts a novel and reliable approach to determine new components as well as validate the previously suggested diets of extinct cultures and animals. Furthermore, to our knowledge this represents the first description of the eukaryotic viral diversity found in paleofaeces belonging to pre-Columbian cultures.

## Significance Statement

In the midst of an increasing awareness towards the importance of our microbiome, i.e. the microorganisms associated to our bodies, this study is the first molecular characterization of the endogenous retroviruses present in fecal samples of pre-Columbian cultures. Thus, this study gives us a glimpse of the virome and diets of ethnic groups in the Caribbean before the arrival of Europeans. Results obtained from this study complement previous archaeological, botanical and anthropological analyses with molecular data. Furthermore, viral sequences present in coprolites allow for the detection of diet components that cannot be discovered by conventional archaeological studies. Overall, this study has allowed us to further understand the everyday lives of these ancient Caribbean cultures.

## Introduction

Coprolites (mummified or fossilized feces) have been shown to document indispensable clues concerning paleodiets, diseases and even ancient cooking habits [1–3]. In previous studies, the origins and diets of ancient species were unraveled with the detection of key genes in their fecal DNA [4,5]. Notably, recent advances in sequencing technology have allowed for a high-throughput molecular approach to studying paleofeces, and thus exponentially enhance the amount of information obtained from these rare specimens [6]. For example, microbial DNA isolated from coprolites not only provides valuable information on the fecal and gut microbiome of the extinct host, but can also suggest key diet components of these ancient specimens [7–10].

In contrast, the viral communities present in ancient feces remain largely unknown, possibly because most viral sequences identified in feces belong to modern-day enteric pathogenic viruses; pathogens whose effects on the host’s intestinal system are likely to prevent the formation of coprolites. Despite previous skepticism, recent studies show that DNA from paleofeces can yield further insight in the composition of ancient viral communities (other than pathogenic enteric viruses) within the human gut [11]. However, the information that could be derived from these ancient viromes concerning specific details of the lives of these extinct organisms has barely been explored so far. To this end, the purpose of this study was to determine if diet-related information could be obtained from the viral DNA in coprolites from two pre-Columbian indigenous cultures (i.e. Saladoid and Huecoid) that inhabited Puerto Rico. Previous carbon-dating indicated the sample ages ranged from 1,400 to approximately 540 uncalibrated years before present.

## Materials and Methods

### Sample description

Coprolites previously obtained from three archaeological deposits in Sorcé Estate, located on the Caribbean island of Vieques were used (**Table 1**) [8]. Carbon-dating analysis was conducted in two separate laboratories (Teledyne Isotopes in Westwood, NJ; BETA Analytic, Inc. in Miami, FL), using standard methods. Excavations in La Hueca site, Sorcé Estate, were lead by archaeologists Luis Chanlatte Blaik and Yvonne Narganes Storde of the Archaeological Research Center at the University of Puerto Rico, Rio Piedras Campus. The University of Puerto Rico is an academic institution and as such is not required to obtain special permits for academic research and archaeological excavations. Excavations on private property in Sorcé, Vieques were done with the owner’s consent.

**Table 1:** Description of coprolites analyzed in this study.

Sample ID	Archaeological section	Quadrant	Depth	Culture	Radiocarbon date	
1	YTA-1	I-5	0.06–0.6m	Saladoid	335–395 A.D.	1620–1680 Y.B.P.
2	YTA-2	J-22	0.8m	Saladoid	270–385 A.D.	1630–1745 Y.B.P.
3	YTA-2	M-25	0.4m	Saladoid	230–385 A.D.	1630–1785 Y.B.P.
15	YTA-2	H-21	1.2m	Saladoid	230–385 A.D.	1630–1785 Y.B.P.
8	SV	Z-W	1.8m	Huecoid	Circa 245 A.D.	Circa 1770 Y.B.P.
4	SV	Z-W	2.0m	Huecoid	1300–220 A.D.	715–1795 Y.B.P.
6	SV	Z-M	1.2m	Huecoid	Circa 450 A.D.	Circa 1565 Y.B.P.
12	SV	Z-X	0.6m	Huecoid	470–600 A.D.	1415–1545 Y.B.P.
13	SV	Z-L	0.7m	Huecoid	Circa 385 A.D.	Circa 1630 Y.B.P.
16	SV	Z-37	0.2–0.4m	Huecoid	470 A.D.	1545 Y.B.P.

Archaeological sections, quadrants and depths correspond to the area where the coprolite was found in La Hueca excavation site, Vieques.

### Preventing contamination

Upon excavation, coprolites were individually stored in sterile sample bags until further use. Samples were individually processed inside a class II biosafety cabinet (BSC) used exclusively for ancient DNA analysis. Strategies used for the obtainment of reliable data were implemented [12]. The BSC was routinely cleaned with 70% ethanol and irradiated with UV for 30 min before and after use. Solutions used for DNA extractions were dispensed into single-use aliquots. Previously unused, sterile micropipettes were used; non-disposable equipment were sterilized by autoclave and baked overnight at >100°C. Baked and autoclaved stainless steel utensils were used to separate the inner and outer layers of the coprolites and were sterilized between samples using 70% ethanol. Previously published data comparing the microbial profiles detected in the inner and outer regions of the coprolites was used as an additional control [8].

### DNA extraction

First, the exterior layers of the coprolites were aseptically removed in a class II biosafety hood used strictly for aDNA, and subjected to controls to avoid extant DNA contamination. DNA was isolated using the commercial PowerSoil DNA extraction kit (MO BIO Laboratories, Inc.) Coprolite cores were ground and 5g of each sample were hydrated overnight in sterile C1 buffer at 4°C. In the case of the Huecoid culture, an additional coprolite was needed to obtain sufficient material from the interior of the coprolites. DNA yield was then concentrated to 10ul using standard glycogen precipitation and pooled to one composite for each ethnic group (MixS1 and MixH1).

### DNA sequencing

The two DNA composites were sequenced in a separate laboratory (MR DNA Research Lab, Shallowater, TX) using a non-targeted metagenomic approach. The library was prepared using Nextera DNA Sample preparation kit (Illumina) following the manufacturer's user guide. The initial concentration of DNA was evaluated using the Qubit® dsDNA HS Assay Kit (Life Technologies). Because of too low DNA concentration for both the samples, whole genome amplification was carried out by using REPLI-g Midi kit (Qiagen) followed by Nextera DNA Sample preparation. The linear amplified DNA was purified using PowerClean DNAClean-Up Kit (MO BIO Laboratories) and the concentration was again evaluated using the Qubit® dsDNA HS Assay Kit (Life Technologies) (**S1 Table**). Samples were then diluted accordingly to achieve the recommended DNA input of 50ng at a concentration of 2.5ng/uL. Subsequently, the samples underwent simultaneous fragmentation and addition of adapter sequences. These adapters were utilized during a limited-cycle (5 cycles) PCR in which a unique index was added to the sample. Following the library preparation, the final concentration of the library (**S1 Table**) was measured using the Qubit® dsDNA HS Assay Kit (Life Technologies), and the average library size was determined using Experion (Bio-Rad). The libraries were then pooled in equimolar ratios of 4nM, and 13.5pM of the library pool was sequenced paired end for 500 cycles using the MiSeq system (Illumina).

### Data processing and identification

DNA reads were filtered using MG-RAST [13] default parameters for quality scores and read length. Overlapping pair-end reads were merged using default parameters, however non-overlapping reads were also retained to maximize the amount of information obtained despite possible fragmentation due to taphonomic processes. Screened reads were downloaded and submitted to diagrid.org hub server (Purdue Research Foundation, West Lafayette, IN, July 2014). Translated nucleotide BLASTX and taxonomical BLASTN analyses were done using the BLASTer tool [14] against the non-redundant NCBI database (National Center for Biotechnology Information). Cut off values for functional and taxonomical identification included 85% minimum similiarity and E value ≤1e-15. All identified sequences with the terms ‘virus’ and/or ‘phage’ in the description were then pooled using the *grep*–*i* command in the shell command prompt (Apple OS X) for further evaluation.

### Project data

Sequence data discussed in this study is included in **S1 Dataset**.

## Results and Discussion

To determine if eukaryote viral DNA in coprolites could suggest the dietary components ingested by ancient specimens, we isolated and sequenced DNA of coprolites from two Caribbean pre-Columbian cultures (n = 4 Saladoid; n = 5 Huecoid, see **Table 1** for more details). Although they are believed to be from South American origins, the Saladoid and Huecoid cultures co-inhabited Sorcé, Vieques for over one thousand years. Unfortunately, the extent to which these ethnic groups influenced modern-day Puerto Rican genetic heritage is unkown.

A brief description of the raw sequence data obtained from metagenomic analysis of pooled samples of each culture is included in **Table 2**. Similar to previous studies, while sequences belonging to bacteriophages infecting the intestinal and fecal microbiota were the most abundant in our data, a small number of DNA fragments from eukaryote viruses were also detected (**S2 Table**) [15,16]. Although the bacteriophages detected in this study will be further discussed in another communication, the presence of key bacteriophages already hinted at possible components of these cultures’ diets. For example, *Vibrio* phages were detected in both cultures; these bacteria can be harbored by a variety of marine organisms, such as crustaceans, that have been proposed by archaeologists as diet components of these cultures. In addition, archaean and protozoan viruses were also detected in lesser proportions. To our surprise, several eukaryote retroviral sequences presented strong evidence of the diets for both cultures (**Table 3**). For instance, avian pox, as well as grouper iridovirus and frog virus sequences were present in Saladoid feces, confirming their consumption of birds, fish and amphibians. This was further supported by the detection of DNA sequences similar to those described in *Xenopus sp*. (**S3 Table**). Avian pox viruses (*Poxviridae*), are chordopoxviruses that infect domestic and wild game birds including hawks, seagulls, parrots, canaries, pigeons, hawks and fowl, causing cutaneous, respiratory and gastrointestinal lesions in their host [17]. Of these, the fowlpox virus is one of the most studied, its genome fully sequenced and annotated [18]. On the other hand, Iridoviruses (*Iridoviridae*) only infect invertebrates and poikilothermic vertebrates such as fish, amphibians and reptiles, often causing serious systemic infections and even death [19]. Our results were further supported by the detection of sequences from protozoan parasites infecting similar viral hosts, such as *Histomonas meleagridis*, which infects a wide range of birds, and *Perkinsus marinus*, an oyster pathogen (this will be further discussed in another communication). Caribbean indigenous cultures are known to have indulged in various bird and fish species as part of their diets. However, the consumption of amphibians was, to the best of our knowledge, previously unreported.

**Table 2:** Nucleotide fragments detected via metagenomic analysis of coprolite DNA.

	Saladoid	Huecoid
**Upload: bp Count**	516,602,540 bp	681,336,171 bp
**Upload: Sequences Count**	1,921,436	2,435,635
**Upload: Mean Sequence Length**	268 ± 63 bp	279 ± 73 bp
**Upload: Mean GC percent**	59 ± 11%	53 ± 9%
**Artificial Duplicate Reads: Sequence Count**	934,579	553,487
**Post QC: bp Count**	159,855,500 bp	369,726,314 bp
**Post QC: Sequences Count**	769,068	1,672,958
**Post QC: Mean Sequence Length**	207 ± 99 bp	221 ± 100 bp

Includes base pair (bp) and sequence count before and after running data through quality control filters in MG-RAST software.

**Table 3:** Viral sequences detected as components of the Saladoid and Huecoid diets.

	Proviral sequence detected	Corresponding gene identification	Virus host organism	Previous osteological, archaeofaunal and botanical results
**Vertebrates** ^a^	Moloney murine leukemia virus	reverse transcriptase	*Mus musculus (rodent)*	*Isolobodon portoricensis* (hutia)
	Murine endogenous retrovirus	retrotransposable element ORF2	*Mus musculus*	*Heteropsomys insulans* (spiny rat)
	Bat endogenous retrovirus	polymerase polyprotein	*Pteropus alecto*	ND
	Tiger frog virus	thymidylate synthase	Frog	ND
	Grouper iridovirus	unknown protein	*Epinephelinae* (fish)	*Epinephelinae*
	Avian pox virus	HAL3 domain	Fowl, parrots, and other birds	*Pelicanus occidentalis* (among many other birds)
	Monkey endogenous retrovirus	H element-like protein	Marmosets	ND
**Invertebrates** ^a^	ND^c^			*Cardisoma guanhumi* (crab)
	ND			*Gecarcinus spp* (crab)
	ND			*Donax denticulatus* (oyster)
	ND			*Cittarium pica* (gastropod)
	ND			*Pelurodonte caracolla* (land snail)
	Mulberry endogenous retrovirus	polymerase polyprotein, transposon TNT 1–94	*Morus notabilis* (berry fruit)	ND
	ND			*Psidium guajava* (guava fruit)
**Plants** ^b^	ND			*Zea mays* (corn)^d^
	ND			*Ipomoea batatas* (sweet potato) ^d^
	ND			*Cassava spp* (yucca)
	ND			*Capsicum spp* (pepper) ^d^

Viral sequences detected in this study are compared to diet components previously suggested by osteological, archaeofaunal and botanical studies of pre-Columbian cultures in Puerto Rico and Vieques.

^a^Previously suggested diet includes observations by the Spanish during there interactions with Caribbean indigenous cultures [20,21], as well as osteological and archaeofaunal analyses of conch shells, teeth and bones obtained from archaeological deposits in Sorcé, Vieques and Puerto Rico [22–25].

^b^Plants suggested as diet components include those proposed by archaeologists as well as plant remains recently identified by paleobotanists through microscopy analysis [26,27].

^c^ND none detected.

^d^Although these diet components were not detected in this shotgun metagenomic analysis, previous *18S rRNA* studies determined their presence in these coprolites [8].

Overall, our results overlap synergistically with those previously suggested by osteological and botanical findings associated with these archaeological deposits and other pre-Columbian cultures in the Caribbean [27,28]. Notably, the majority of the eukaryote viruses identified in this study were known retroviruses, strongly suggesting the presence of proviruses and/or the lateral acquisition of genes during the host-parasite interaction. This was no surprise as viral “junk” DNA is known to occupy approximately 4.8% of the human genome; in contrast, eukaryote protein-coding genes comprise only 3% of the same genome [29]. Furthermore, the presence and expression of horizontally-acquired genes are now known to be common in host-parasite interactions involving vertebrates and invertebrates [30,31]. Endogenous retroviruses (ERV) in particular are viral gene remnants that seem to have integrated into the host genome after infection [32]. ERVs are passed onto the host’s progeny and may persist for over a hundred million years in the germline; therefore, they serve as an excellent snapshot of a species’ history of infection [33,34]. In addition, within the eukaryote genome ERVs are subject to lower mutation rates as opposed to non-retroviral RNA viruses [35].

We observed DNA sequences from various endogenous eukaryote retroviruses, such as those infecting humans (HERVs), as well as those infecting rodents, flatworms, nematodes, bats and fungi, in coprolites from both cultures (**Table 3**). Pre-Columbian indigenous cultures in the Caribbean and other regions of the world have previously been reported to eat cooked and possibly lightly cooked meat [20], as well as roasted, desiccated and even raw vegetables [36]; such eating habits could explain the presence of detectable remnant eukaryotic DNA in their feces. Osteological studies suggest that rodents such as *Isolobodon portoricensis* (hutía) and *Heteropsomys insulans* (spiny rat) were normal diet components of the Saladoid and Huecoid, among other Caribbean cultures [37]. Furthermore, evolution and colonization studies of the Antilles suggest the presence of a much higher diversity of rodents (among other animals) in the Caribbean islands than what was previously believed [38]. It was therefore no surprise that we detected sequences similar to those of viruses infecting the Murinidae family, some of these rodents’ few relatives sequenced to date (**Table 3**). This was further supported by the detection of rodent DNA in these samples, particularly similar to *Octodon degus* (**S3 Table**). Although bats endemic to the area at that time were often symbolized in their religious artifacts, until now it remained uncertain if these animals, such as *Phyllonycteris major* for example, were a part of the Saladoid and Huecoid diets. The detection of bat ERVs in feces from both cultures strongly suggests their consumption. Finding oncogenic retroviral sequences in these coprolites opens the possibility of looking at viral pathogens as additional factors that could have aided in the sudden decline in the populations of certain rodents in this island, such as the hutía and the spiny rat, which to date is commonly thought to have become endangered as a result of weather patterns or over-predation. Although natives of most Caribbean islands fed on the “hutía”, it is peculiar that these rodents only went extinct in Vieques and Puerto Rico, but still thrive in nearby islands. Similarly, bones of the Puerto Rican spiny rat are rarely found in archaeological excavations on the island, indicating that these organisms were not commonly hunted by these cultures, however they are also believed extinct.

Ancient diets have been reconstructed using a variety of methods. For example, morphological and chemical analyses of the bacterial residues found in a dinosaur’s coprolite which clearly suggested an herbivorous diet [39]. Similarly, transient DNA in the gut (and feces) has also been studied in order to detect key genes associated to the host diet, such as chloroplast-specific sequences, for example [40,41]. However, these analyses have yet to include the gut virome, an indispensable and highly populated community of the gut biome. This study clearly demonstrates the novel information that may be obtained from the fecal virome and provirome of ancient organisms through the use of a shotgun sequencing analysis as opposed to selective gene-screening methods. For instance, the detection of over forty DNA fragments corresponding to proteins encoded by *Capsaspora owczarzaki*, a snail symbiont, strongly suggests a previously unknown yet common diet component of the Saladoid culture (data not shown). This will be further discussed in another communication. Also, the presence of retroviral DNA from marmoset New World monkeys may support the hypothesis of ancient organic trade between Caribbean and South American cultures [21]. In addition, as shown here the presence of host-specific viral and proviral DNA can help validate hypotheses generated by other methods and disciplines studying the diets and customs of ancient specimens. It is of high interest that sequences belonging to a European plant virus were found (*Morus notabilis*); and this finding opens up more questions than it answers. Although highly hypothetical, and perhaps speculative, these sequences may belong to a Caribbean, pre-Columbian virus that was later introduced to Europe capable of infecting the mulberry tree. This demonstrates one limitation of relying solely on DNA sequences to answer complex questions. It remains to be seen if this hypothesis holds for other unexpected sequences found. Notably, no DNA sequences belonging to either *Cassava* spp., *Ipomea* spp. and *Zea* spp. or their viruses were found in these coprolites when using metagenomic sequencing, in spite of them being staples for these cultures. Curiously, our group had previously detected *Zea* and *Ipomea* sequences when using *18S rRNA* sequencing. This could be due to the DNA amplification steps used in microbial profiling methods, which could presumably target low abundance DNA. This observation also points to the limitations intrinsic to each method, particularly those common in metagenomic analyses without the pre-amplification of DNA. A possible conclusion may be that these foods were thoroughly cooked prior to consumption, thus although consumed in large amounts, the target DNA may be degraded and thus limiting the possibility for detection by metagenomic analyses. Interestingly, DNA of the pea aphid Acyrthosiphon pisum was found in Huecoid feces, suggesting the consumption of these legumes (**S3 Table**). Some legumes are endemic to the Caribbean islands, others are often found in South America, where the Saladoid and Huecoid cultures are thought to have originated [23,35].

## Conclusions

Advances in metagenomics have increased our perception of the highly complex host-microbiome interactions ocurring within a holobiont. Similarly, the effects certain diets may induce on the dynamics of the gut microbiota are still being determined. This study demonstrates the novel approach of demonstrating the usefulness of the virome and provirome in ancient specimens for the reconstruction of the diets of extinct cultures. In fact, the apparent potential and abundance of proviruses in coprolites point to their importance in future molecular paleovirological studies. Our results complement the currently limited molecular data available on the diets of indigenous cultures prior to the arrival and colonization by Europeans [42]. Fan-Ng, et al have also shown that viral sequences found in paleofeces of extinct animals can be used as a means of taking a peek at the past and an opportunity to reconstruct their diet [43]. Our study and previously published results are also slowly changing the apparent dogmata on the half-life of DNA and its resilience over time [44,45]; however, DNA resiliency studies have been done using purified DNA, thus shedding little light on the survival of intracellular DNA during taphonomic processes over millennia [46]. It is clear from our studies, though, that intracellular DNA is in fact more resilient than previously expected.

## Supporting Information

S1 Table DNA and library concentrations. ^1^Indicates whole genome amplified and purified DNA. Reported size includes sequencing adaptors, measuring approximately 120bp each.
(DOCX)

S2 Table Virus genes detected by translated-nucleotide query of coprolite DNA from both cultures. ^1^gi| NCBI gene identification number. ^2^Identification code of the corresponding DNA sequence detected in Huecoid (H-A4LNU…) and Saladoid (S-A4LNU…) coprolite samples. (See **S1 Dataset** for complete DNA sequences).
(DOCX)

S3 Table Description of eukaryote diet-associated genes detected after conducting a translated-nucleotide query of coprolite DNA from both cultures. ^1^gi| NCBI gene identification number. ^2^Identification code of the corresponding DNA sequence detected in Huecoid (H-A4LNU…) and Saladoid (S-A4LNU…) coprolite samples. (See **S1 Dataset** for complete DNA sequences).
(DOCX)

S1 Dataset Fasta format file containing the trimmed, post-QC ancient DNA sequences described in this study. These sequences correspond to the genes identified through blast query search.
(DOCX)
