# Supplementary material for: Retroviral DNA Sequences as a Means for Determining Ancient Diets
Source: PLoS One. 2015 Dec 14;10(12):e0144951. doi: 10.1371/journal.pone.0144951 (PMC4682816; doi:10.1371/journal.pone.0144951)
Supplement: S1 Table — 1Indicates whole genome amplified and purified DNA. Reported size includes sequencing adaptors, measuring approximately 120bp each. (DOCX) [file pone.0144951.s001.docx]

**S1 Table. DNA and library concentrations.**

| Sample | DNA concentration (ng/uL)^1^ | DNA library concentration (ng/uL) | Average library size (bp) |
| --- | --- | --- | --- |
| MixS1 | 85.20 | 7.70 | 1230 |
| MixH1 | 83.80 | 12.30 | 1550 |

^1^Indicates whole genome amplified and purified DNA. Reported size includes sequencing adaptors, measuring approximately 120bp each.
