# Supplementary material for: Retroviral DNA Sequences as a Means for Determining Ancient Diets
Source: PLoS One. 2015 Dec 14;10(12):e0144951. doi: 10.1371/journal.pone.0144951 (PMC4682816; doi:10.1371/journal.pone.0144951)
Supplement: S2 Table — 1gi| NCBI gene identification number. 2Identification code of the corresponding DNA sequence detected in Huecoid (H-A4LNU…) and Saladoid (S-A4LNU…) coprolite samples. (See S1 Dataset for complete DNA sequences). (DOCX) [file pone.0144951.s002.docx]

**S2 Table**. Description of virus gene fragments detected after conducting a translated-nucleotide query of coprolite DNA from both cultures.

|  | Proviral sequence detected | Corresponding gene identification | NCBI Blast result  gene identification^1^ | Corresponding DNA sequence detected in coprolites^2^ |
| --- | --- | --- | --- | --- |
| Vertebrates | Moloney murine leukemia virus | reverse transcriptase | gi\|15988472 | H-A4LNU:1:1112:26766:20583 |
|  | Murine endogenous retrovirus | retrotransposable element ORF2 | gi\|60392921 | H-A4LNU:1:2114:15641:14324 |
|  |  |  | gi\|7522108 | S-A4LNU:1:2105:12198:14082 |
|  |  |  |  | S-A4LNU:1:2106:20870:17837 |
|  |  |  |  | S-A4LNU:1:2113:18531:9667 |
|  |  |  |  | S-A4LNU:1:1104:18122:9588 |
|  |  |  |  | S-A4LNU:1:2104:15729:8086 |
|  |  |  |  | S-A4LNU:1:2110:2675:19022 |
|  |  |  |  | S-A4LNU:1:2110:13777:15202 |
|  |  |  |  | S-A4LNU:1:1108:19302:8263 |
|  |  |  |  | S-A4LNU:1:2112:28647:12352 |
|  |  |  |  | S-A4LNU:1:1113:11779:7652 |
|  |  |  |  | S-A4LNU:1:1112:3317:16417 |
|  |  |  |  | S-A4LNU:1:1106:13720:8316 |
|  |  |  |  | S-A4LNU:1:1108:5433:14444 |
|  |  |  |  | S-A4LNU:1:1108:19302:8263 |
|  |  |  |  | S-A4LNU:1:1109:26411:13916 |
|  |  |  |  | S-A4LNU:1:1113:25773:13002 |
|  |  |  |  | S-A4LNU:1:2110:22904:12538 |
|  |  |  |  | S-A4LNU:1:2104:20548:4355 |
|  |  |  |  | S-A4LNU:1:2114:28971:10847 |
|  |  |  |  | S-A4LNU:1:1102:11143:8448 |
|  |  |  |  | S-A4LNU:1:1107:13012:9571 |
|  |  |  |  | S-A4LNU:1:1106:15913:9320 |
|  |  |  |  | S-A4LNU:1:1114:21164:2517 |
|  |  |  |  | S-A4LNU:1:1112:11072:14753 |
|  |  |  |  | S-A4LNU:1:1110:10464:25355 |
|  |  |  |  | S-A4LNU:1:1113:25652:10479 |
|  |  |  |  | S-A4LNU:1:2111:13229:13285 |
|  | Bat endogenous retrovirus | polymerase polyprotein | gi\|431914787 | H-A4LNU:1:1106:14735:20920 |
|  |  |  |  | S-A4LNU:1:2105:16425:18793 |
|  |  |  |  | S-A4LNU:1:2109:18154:11273 |
|  |  |  |  | S-A4LNU:1:1110:23150:7993 |
|  |  |  |  | S-A4LNU:1:2109:14197:8517 |
|  | Tiger frog virus | thymidylate synthase | gi\|18656511 | S-A4LNU:1:2101:11587:11252 |
|  | Grouper iridovirus | unknown protein | gi\|56418269 | S-A4LNU:1:1108:9314:13541 |
|  | Avian pox virus | HAL3 domain | gi\|9634784 | S-A4LNU:1:2106:13882:25479 |
|  | Monkey endogenous retrovirus | H element-like protein | gi\|1217671 | S-A4LNU:1:1113:10929:20186 |
| Invertebrates | *Clonorchis sinensis* endogenous retrovirus | Polymerase polyprotein from transposon 17.6 | gi\|35833622 | H-A4LNU:1:1114:17137:21548 |
|  |  |  |  | H-A4LNU:1:2111:20451:14710 |
|  |  |  |  | H-A4LNU:1:1110:21317:13784 |
|  |  |  |  | S-A4LNU:1:1101:14085:3628 |
| Plants | Mulberry endogenous retrovirus | polymerase polyprotein,  transposon TNT 1-94 | gi\|587900079 | S-A4LNU:1:1104:25363:18971 |

^1^gi| NCBI gene identification number.

^2^Identification code of the corresponding DNA sequence detected in Huecoid (H-A4LNU…) and Saladoid (S-A4LNU…) coprolite samples. (See fasta file for complete DNA sequence).
