# Supplementary material for: Retroviral DNA Sequences as a Means for Determining Ancient Diets
Source: PLoS One. 2015 Dec 14;10(12):e0144951. doi: 10.1371/journal.pone.0144951 (PMC4682816; doi:10.1371/journal.pone.0144951)
Supplement: S3 Table — 1gi| NCBI gene identification number. 2Identification code of the corresponding DNA sequence detected in Huecoid (H-A4LNU…) and Saladoid (S-A4LNU…) coprolite samples. (See S1 Dataset for complete DNA sequences). (DOCX) [file pone.0144951.s003.docx]

**S3 Table**. Description of eukaryote diet-associated genes detected after conducting a translated-nucleotide query of coprolite DNA from both cultures.

| Host DNA sequences detected | Corresponding gene sequence | NCBI Blast result gene identification^1^ | Corresponding DNA sequence detected in coprolites^2^ |
| --- | --- | --- | --- |
| *Acyrthosiphon pisum*  (pea aphid) | PREDICTED: influenza virus NS1A-binding protein | gi\|193618018 | H-A4LNU:1:2101:23195:10718 |
|  |  |  | H-A4LNU:1:2111:8079:4546 |
|  |  |  | H-A4LNU:1:1101:12673:1875 |
| *Xenopus sp.* | ubiquitin specific peptidase 7 | gi\|189217677 | H-A4LNU:1:2107:18445:8332 |
|  |  |  | H-A4LNU:1:1111:10292:12247 |
|  |  |  | H-A4LNU:1:2102:10614:4357 |
|  |  |  | H-A4LNU:1:2104:25548:7151 |
| *Octodon degus*  (rodent) | PREDICTED: Friend virus susceptibility protein 1 | gi\|507645756 | H-A4LNU:1:2102:23814:25938 |
| *Octodon degus*  (rodent) | PREDICTED: Friend virus susceptibility protein 1 | gi\|507681742 | H-A4LNU:1:2106:18947:13213 |
|  |  |  | S-A4LNU:1:1103:12506:5820 |
|  |  |  | S-A4LNU:1:1104:21289:25308 |
|  |  |  | S-A4LNU:1:2112:14158:11077 |
|  |  |  | H-A4LNU:1:1103:7510:6803 |

^1^gi| NCBI gene identification number.

^2^Identification code of the corresponding DNA sequence detected in Huecoid (H-A4LNU…) and Saladoid (S-A4LNU…) coprolite samples. (See fasta file for complete DNA sequence).
