# Supplementary material for: Retroviral DNA Sequences as a Means for Determining Ancient Diets
Source: PLoS One. 2015 Dec 14;10(12):e0144951. doi: 10.1371/journal.pone.0144951 (PMC4682816; doi:10.1371/journal.pone.0144951)
Supplement: S1 Dataset — These sequences correspond to the genes identified through blast query search. (DOCX) [file pone.0144951.s004.docx]

**Supplementary Data**

*Huecoid DNA*

> H-A4LNU:1:1112:26766:20583

GTGTTACCTCAGGGATTCCAAGACAGCCCCTGCTTATTTGGCCAGGCATTCTCAAAAGACCTCTTTGAGTTCCCTTATCCTCAGGTTAAAGCTTTACAATACGTAGATGACATTCTCCTCTGTGCCATAACTGAGGCAATCTCTCAGGAGGGCAGTAAGGGTCTTCTTAATTTTCTGGCTAACAGAGGATATAAGATTTCAAAGCCTAAAGCTCAGCTCTGTCAGACTTCAGTGAAGTACCTAGACCTGGTCTTTCAGAGAGGACCAAGGCATTGGGTGAAGAAAGGATTAAGCCCATCTTCTTTTTTCCCCTCTCCAAAACC

>H-A4LNU:1:2114:15641:14324

CATACACACACACACACACACAAGCACACACACACACACACACAAATAGATTCCATGTTCATAGAAGAATCAGCACTGTTCAAATGTTCATACTGCCCAAAGAAATATACAGATTTAATGCAATTTCTATCAAAATACCAATTATGTTCTCCTCAGAAATAGAAAAAATTATTTTCAAATGTATATGGAACCACAAATGACCCAGAATAGCCAAAGCTACACTAAGCAGAAAGAAGAAAACTGGAGGGATTGCATTACCTGACTTTGAGTTATACTACAGAGCTATCATAATCAAAATGGCATGGTACTGGCATGAAAATGAACAAATAATTAATGGAACAGAATAGAGAACCCAGAAGTACATCCATACATCTACAGTGAACTCATTTTCGACAAGTTTCCAAGAACATACATTTGAGAAAGGACTGTCTCTTCAACAAATGGTGCTGGGAAAATTGGATATCCACATGTAGAAGAA

>H-A4LNU:1:1106:14735:20920

GTATTCACCCAAAGGAAAACAAGTCATTATATCAAAAACACACCTGCACTCATATGTTTATTACAGCACCATTCACAATAGCAAAGATACGGAGTCAACCCAGGTGCTCATCGACAGGTGATTGGATACAGAAAATGTGATTTATGTGTACAATGGAATCATATTCAGCCATGCAAAAGAATGAAATTCTATCTTTTGCAGCAACATGGATGAAACTGGAGGCCATTATCTTAAGTCAAATAACTGAGAAAAAGAACGTCAATTACCACGTGTTCTCACTCATAAGTGGGAGCTAAATAATATGTCCACATGGACATAGAGAGTGGATTAATAGACACTGGAGATACAAAAGGGTGAGAGAGTGGGAGGATGGTGGAGAATGAGAAATTACTTAATAGGTACCATG

>H-A4LNU:1:1114:17137:21548

TGATAGTAGAACGTGTGTCGGTCTACTATAGTTTTTAGACTTCGTGTGTCGAGTCTATAAAAACCGTGTGTCGGTTTTACTTCTTTCTATCTCCTTAGATATAATCGTTTGATTATCAAAACTCAACATTAGCATATACTGTATCTCTTTTCTTAAACATACGTCTTCCCTGCTATTAGTTATCTGTATCTGTTTATTAGAGTTAGGATAGCTGAATGGTTTTCGACGTTAGATCTCGCCAGTGGATACTGGCAAGTTGAAGTCGTGCCATCGGACAGACCAAAGACAGCATTCATCACCAGGTATGGCATTTATGAGTTCAATGTTATGCCGTTTGAGCTTACCAATGCGCCTGCCACCTTCCAACGCCTCATGAACACTGTGCTCGCTGGATCCTTATAGGAT

>H-A4LNU:1:2111:20451:14710

GCATTACTGTGAACTCGTAGGTGCTATATTTAGTAATAAATGCCATTTTTTCTCAGTCCTCTTTTTTCATTTTAATTTGCCAGTATCCACTAGCAAGATTAATAGTAGAAAACCATTTAGCATTACCCATCATAGATAATACTTCATCAATGACTAGTAAAGGATAACTATCCTTTTTAGTTATTTTGTTTAACTTTCGATAGTCTACACACATTCGTAACTTTCCATTATTTTTAGGTACTAATACTATAGGCGAAGCCCATGGACTTGAAGATTCTTGGATAATT

>H-A4LNU:1:1110:21317:13784

TTTTATAGGACATCGCTGCATGAACATTAATTTATCGGGCAGCAAATTGCTCAGTTGTTACATCAAGGATTAATTCAATCATCGACTAGCTCGTAGGCCTCGCCAGTGGTTCTGGTACGCAAGAAAAATGGAAAGCTCAGACTTTGTGTAAATTATCAAAAATTAAATTCGGTAACTTTGAAAGACAGTTATCTGCTACCAAGGATCGATGACCTTCTGGATGCGTTAGGATAAGCTGAATGGTTTTCGACGTTAGATCTCGCCAGTGGATACTGGCAAGTTGAAGTCGTGCCATCGGACAGACCAAAGACAGCATTCATCACCAGGTATGGCATTTATGAGTTCAATGT

>H-A4LNU:1:2101:23195:10718

GTACTCCACTCCCCTTGCCTGCCGTCAGCACTCACGTCGAGCACCTCTACCGTAGCTAGATCGCCTCCCGAGTCAGAGGTGCCATCCCAACCACCAATCACGTAGACCCGGTTGCCTTGTACCGCAATGGCAAAGTCGGAGCGCGGGGTGGGCATAGACGGGCCGGAGGCCCACTCTCCGGTGTTTATATCGTAGACTTCGACGGTGCTGAGCACGGTGCCACTCTTCAGGATGCCCTGTCCTCCATTGCGGCCTCCAATGGCATAGAGCTTCTTACCGACGGCAGCTAGACCCAGCCCGGCGCGTGGCGTAGGCATCGGCCTGAGGGTTTGCCATTTATCTTTCCTGGTATCATAAGCCTCGAAGGTGTTCAAA

>H-A4LNU:1:2111:8079:4546

ACCTTACAACATCGCCATTTTGAACACCTTCGAGGCTTATGATACCAGGAAAGATAAATGGCAAACCCTCAGGCCGATGCCTACGCCACGCGCCGGGCTGGGTCTAGCTGCCGTCGGTAAGAAGCTCTATGCCATTGGAGGCCGCAATGGAGGACAGGGCATCCTGAAGAGTGGCACCGTGCTCAGCACCGTCGAAGTCTACGATATAAACACCGGAGAGTGGGCCTCCGGCCCGTCTATGCCCACCCCGCGCTCCGACTTTGCCATTGCGGTACAAGGCAACCGGGTCTACGTGATTGGTGGTTGGGATGGCACCTCTGACTCGGGAGGCGATCTAGCTACGGTAGAGGTGCTCGACGTGAG

>H-A4LNU:1:1101:12673:1875

CGCCTGGTGGTCCGCTGCAACCTTACAACATCGCCATTTTGAACACCTTCGAGGCTTATGATACCAGGAAAGATAAATGGCAAACCCTCAGGCCGATGCCTACGCCACGCGCCGGGCTGGGTCTAGCTGCCGTCGGTAAGAAGCTCTATGCCATTGGAGGCCGCAATGGAGGACAGGGCATCCTGAAGAGTGGCACCGTGCTCAGCACCGTCGAAGTCTACGATATAAACACCGGAGAGTGGGCCTCCGGCCCGTCTATGCCCACCCCGCGCTCCGACTTTGCCATTGCGGTACAAGGCAACCGGGTCTACGTGATTGGTGGTTGGGATGGCACCTCTGACTCGGGAGGCGATCTAGCTACGGTAGA

>H-A4LNU:1:2107:18445:8332

GCTTCACACACACTGCAGGAGCAATGGGGTATCTGCAAGATCCCCTCAACCTTGAACTGATCTTTCAGGTGGGATTCAAAGAAGCACACAGGCTACGTCGGCTTAAAGAATCAGGGAGCGACTTGTTACATGAACAGCCTGCTACAGACGTTATTTTTCACGAATCAGCTACGAAAGGTAAATGGATAGTGTTCCTCTCCTGCAGCCTTCCGCTCACCCTTTCTTCTCAGGTATATCAGAGCAAAGTCCTCTCAACAAACATGGCGTCTCCCTC

>H-A4LNU:1:1111:10292:12247

TGTTTGTTGAGAGGACTTTGCTCTGATATACCTGAGAAGAAAGGGTGAGCGGAAGGCTGCAGGAGAGGAACACTATCCATTTACCTTTCGTAGCTGATTCGTGAAAAATAACGTCTGTAGCAGGCTGTTCATGTAACAAGTCGCTCCCTGATTCTTTAAGCCGACGTAGCCTGTGTGCTTCTTTGAATCCCACCTGAAAGATCAGTTCAAGGTTGAGGGGATCTTGCAGATACCCCATTGCTCCTGCAGTGTGTGTGAAGCAATCTGACTCAAGGTCAGCCTTAAGGAAACCGAGGCCAGGAACACTTATCACACAGCATCCTTCAATGGTCACCGATTCCCTTCTGCTTGCTCCAGTGTGTGAACTGGTGCAAATGCAACCCCCACATTCGCACAGCC

>H-A4LNU:1:2102:10614:4357

GTCCCAGCACTGGGGAATGAAAACGCAGCATGAACTAGCAGCCATCTGAGGAGACAGGGCCAGGGGAGGGAGACGCCATGTTTGTTGAGAGGACTTTGCTCTGATATACCTGAGAAGAAAGGGTGAGCGGAAGGCTGCAGGAGAGGAACACTATCCATTTACCTTTCGTAGCTGATTCGTGAAAAATAACGTCTGTAGCAGGCTGTTCATGTAACAAGTCGCTCCCTGATTCTTTAAGCCGACGTAGCCTGTGTGCTTCTTTGAATCCCACCTGAAAGATCAGTTCAAGGTTGAGGGGATCTTGCAGATACCCCATTGCTCCTGCAGTGTGTGTGAAGCAATCTGACTCAAGGTCAGCCTTAAGGAAACCGAGGCCAGGAACACTTATCACACAGCATCCTTCAATGGTCACCGATTCCCTTCTGCTTGCTCCAGTGTGTGAACTGGTGCAAATGCAACCCCCACATTCGCACAGCCC

>H-A4LNU:1:2104:25548:7151

GGTTACAAAGTAGGTGTCCCAGCACTGGGGAATGAAAACGCAGCATGAACTAGCAGCCATCTGAGGAGACAGGGCCAGGGGAGGGAGACGCCATGTTTGTTGAGAGGACTTTGCTCTGATATACCTGAGAAGAAAGGGTGAGCGGAAGGCTGCAGGAGAGGAACACTATCCATTTACCTTTCGTAGCTGATTCGTGAAAAATAACGTCTGTAGCAGGCTGTTCATGTAACAAGTCGCTCCCTGATTCTTTAAGCCGACGTAGCCTGTGTGCTTCTTTGAATCCCACCTGAAAGATCAGTTCAAGGTTGAGGGGATCTTGCAGATACCCCATTGCTCCTGCAG

>H-A4LNU:1:1103:7510:6803

CAGCTGGACTACTGACATGCATGAGTGCTGTCTTAGTCAGCACTCTTCAGCTCTTCAAAGATGCAGGTGCTCCCCTGACAAATCTATTTCACAAGGCATTGGTCAAGACTATATCTTCTGGACCTTCCCAGCTGGGATGAGTAGGTCAAAAGTGACTAATCCACTCCACCATCCCCATCTCCCCAAGCCTTTGGATCCCCTCCTCTACATTAAGCTAAGGGAGATCAGGCATTTCCAGCTCACTCACAGTGGGCCATCTTTTAATCCATAGTTCAGTTAATCAAGCAAATAGACAATTAGAACCTTTTTGAACAAATAAATGTATAAAAAATGTCAGGTAGTGAGAAGTGCAATGAAGAAAATAACACAAGATAAGGCTTTCAAGAACAAGGCTGCTGTATTATACAGGAG

>H-A4LNU:1:2102:23814:25938

CTTCTGGACCTTCCCAGCTGGGATGAGTAGGTCAAAAGTGACTAATCCACTCCACCATCCCCATCTCCCCAAGCCTTTGGATCCCCTCCTCTACATTAAGCTAAGGGAGATCAGGCATTTCCAGCTCACTCACAGTGGGCCATCTTTTAATCCATAGTTCAGTTAATCAAGCAAATAGACAATTAGAACCTTTTTGAACAAATAAATGTATAAAAAATGTCAGGTAGTGAGAAGTGCAATGAAGAAAATAACACAAGATAAGGCTTTCAAGAACAAGGCTGCTGTATTATACAGGAGGCCTGGG

>H-A4LNU:1:2106:18947:13213

GTTATGATCAACCCAGAGCACCAAAATGCTGAATGGCATGCCAGCTGGACTACTGACATGCATGAGTGCTGTCTTAGTCAGCACTCTTCAGCTCTTCAAAGATGCAGGTGCTCCCCTGACAAATCTATTTCACAAGGCATTGGTCAAGACTATATCTTCTGGACCTTCCCAGCTGGGATGAGTAGGTCAAAAGTGACTAATCCACTCCACCATCCCCATCTCCCCAAGCCTTTGGATCCCCTCCTCTACATTAAGCTAAGGGAGATCAGGCATTTCCAGCTCACTCACAGTGGGCCATCTTTTAATCCATAGTTCAGTTAATCAAGCAAATAGACAATTAGAACCTTTTTGAACAAATAAATGTATAAAAAATGTCAGGTAGTGAGAAGTGCAATGAAGAAAATAACACAAGATAAGGCTTTCAAGAACAAGGCTGCTGTATTATACAGGAG

*Saladoid DNA*

>S-A4LNU:1:2109:14197:8517

GGTTCAGGCTGCTGTGCAAGCTGCTCTGCCACTTGGGCCATATGACCCAGCAGATCCAATGGTGCTTCAGGTGTTAGTTGCAGATAGGGATGCTGTTTGGAGCCTTTGGCAGTCCCCATAGTTGAATCACAGTGGAGGCCACTAGGATTTTGGAGCAAGGACCTGCCATCTTCTGCAGATAACTATTCTCCTTTTGAGAGACAGCTCTTGGCCTGTTACTGGGCTGTGGTGGAAACTGAACATTTGACTATGGATCATGAAGTCACCATGTGACCTCAACTGCCTATCATGAACTGGGTGCATTCTATCCCATCTAACCATAAAGTGGGTCATGCACAGCAGCATTCCATCATAAATGGAAGTGGTATATGTTACCAGGCTTGAGCAGGTCCTGAAGGCACAAGTAAGTTACATGAGGAAGTGGCGGAAGTGCCCGTTGTCTCCACTCCTGCCACCC

>S-A4LNU:1:2105:12198:14082

AAATCAAGAGAGATCAGGCATTGGCTAATTAATCACGGTGTTCCCAGAAGTGAAATTGATAGGAAGCCTACTATATTTCTACTTAATTTATATAAGCAGAAATCTTCAGGTTGAATGGACAAAATACTAATTTGAATTATAGAAACAGAGAATTATGGCCCCTTAATCAATTTCCAGACTTGAGCAAGTTTACACACCCAGAACCCCTTGAATGAAGGGGAGGCTGGGTCCCCTTAAGGAAGGACCCCAATACAATACTGACAATTTATGCTGCTAATCTTTCTCCCATCCTTCCCCAAGGAGACCTCCGGCCTTTTATCAGGGTAACTGTGCATTTTGAGAAAGGGAAATGATCAGATATTTCGGGGACTACTGGACACTGGCTCTTAGCTGACGTGGATTCCAGGGGACTCAAAATGTCATTGTGATCCTTCAGTTAAAGTAGGGACTTATGG

>S-A4LNU:1:2106:20870:17837

TTCACTATTACCTTTAGGGACCCACTAGCAAAATTTTTGCTTCCTGTTCCTGCAACCTCTTCTCTGCTGGCCTAGAGGTCTTAGTTCCAAAGGGAGAAGTTCTTCCATCAGGAGACACAAAAATGATTCTTTTGAACTGGAAGTTAAGACTTCCAGCCATTTAGGGCTCCTCATGACTCCAAATCAATCAACAGGCAAAGAAAGGAGTTTTTGTACTGCCTGGAGTGAGTGATCCTGACTTCCAAGGGGAAACTGAACTGCTACTCCACTATGGATGTATGGAATGTAGGAGATTCCTTAGCATGTCTCTTAGTGTCACCATGCCCTGTGATTAATGTCCATGAAGAGTGACCCAATCCAGGAA

>S-A4LNU:1:1104:18122:9588

GTTCCATGCATACTCTTCCTTACCTCCATTATGGAATAGCAGACTGATTTCATCTTGACAGTCCAGGTCAATCAGCCCAGCCAACACTGTAAATCCCTTCTTATCCTGTTGACTTTAGGATAGGAGCAGCCCAAAGTGTCCAGGTGGAAATCTTAACTTCCAGTTTAATGGAAACTTTGTTGTGTTTCCTAGTGGCAGCATTCCTCCCTCTGGAACTAAGACCTCTAGGCCAGGAGAACATAATGTCAGGGAAACAGGAAGCAAACATTTGCTAGTGGATTACTAGCGGTGATGGTGAGTGGTGTCACTTCCATTTCCACCCCTTGATTCCTGGACCCGTGAATCCTTGCTATGGGAGAAATAGTACCATATATTGAACACTGATTCAGAGCATACATGGTCTTCTAGAGAACTTTGCCCCAGCCCTGCAAAGTATTGTCATCTAGTTGGCGTTGTAATTGTGACTTCAAAAGGCCATTACACCGTTCTATC

>S-A4LNU:1:2104:15729:8086

GCCTTGGTCAGAGGTAATGCTGTGTGGAATACCATGATGGTGGATAAGGCATTCCATGAGTCCACAGATGGTAGTCTTGGCAGAAGTATTGCATGCAGAATAGGTAAACCCATATCTGGAGTAAGTGCCTATTCCAGTGAGGACAAACCTCTGCCCTTTCCATGATGGAAGAGGTCCAATATAATCAACCTGCCAC

>S-A4LNU:1:2110:2675:19022

ACTGATAATTTATGCAGTGAATCTTTCTCCCATCCTTCCCCAAGGACACCTCTGGCTTTTTACCAGGGTAACTGTGCACTGGAGAATGGGAAATGATCGGACATTTTGGGGACTACTGGACACTGTCTCTGAGCTGATGTTTATTCCAGGGAACCCAAAACATCATTGTGGCCCTCCAGTTAAAGTAGGGGCTTATGGAGATCAGG

>S-A4LNU:1:2113:18531:9667

GCCTTATGGACAGGAATGGAGAAAAAGGCATTTTCCAAGTAAATGGCTGCACACCAGGTACCAGGAGACGCGTTAATTTGCTCAAGCAATGAAACCACTTCTGGTACAGCAGCTGCAATTGGAGTCACCACGTGGTTACTGTCTTCTGTGCAGGCCAAATGGGAGAGTTGAACGCAGATGTGGTAGGAATCACCACCCCTGCATCTTTCAAGTCCTTGATGGTAGCACTAATCTCTGCAATCCCTCCAGGGATGCAATATTGTTTTTGATTTATCATTTTTCTAGGTAGAGACAGCTCTGCTTCCATTTGGTCTTTCCCACCACAATAACTCTCACCCTAACAGTCAGGGAGCTAATGTAGGGGTTCTACCAGCTGCTAAGTATGTCTATGCCAATTACCATTCTGGCACTGGGGAAATGACCACAGGATGAGTCTGGGGACC

>S-A4LNU:1:2110:13777:15202

GGACCTGCCATCTTCTGCAGATAACTATTCTCCTTTTGAGAGACAGCTCTTGGCCTGTTACTGGGCTGTGGTGGAAACTGAACATTTGACTATGGATCATGAAGTCACCATGTGACCTCAACTGCCTATCATGAACTGGGTGCATTCTATCCCATCTAACCATAAAGTGGGTCATGCACAGCAGCATTCCATCATAAATGGAAGTGGTATATGTTACCAGGCTTGAGCAGGTCCTGAAGGCACAAGTAAGTTACATGAGGAAGTGGCGGAAGTGCCCGTTGTCTCCACTCCTGCCACCCTGGCTCCTCTCCCCCAGGTTGCACTGATGGCCTCATGGAGAG

>S-A4LNU:1:2112:28647:12352

GTGTGGCAGTGGGCTCATGCTCATGGAATTCACTGGTTTTACCATGTTCCCCATCATCCTGAAGCAGCTGGATTGATAGGACAGTGGAATGGCTTTTTGAAGTCATAGTTACAACGCCAACTAGGTAACAATACTTTGCAGGGCTGGGGTAAAGTTCTCCAGAATACCATTGACTACAGCCCAAGAATCACCATATA

>S-A4LNU:1:1113:11779:7652

GTCTTACCATGTTCCCCATCATCCTGAAGCAGCTGGATTGATAGAACGGTGTAATGGCCTTTTGAAGTCACAATTACAACGCCAACTAGATGACAATACTTTGCAGGGCTGGGGCAAAGTTCTCTAGAAGACCATGTATGCTCTGACTCAGTGTTCAATATATGGTACTATTTCTCCCATAGCAAGGATTCACGGGTCCAGGAATCAAGGGGTGGAAATGGAAGTGACACCACTCACCATCACCGCTAGTAATCCACTAGCAAATGTTTGCT

>S-A4LNU:1:1112:3317:16417

GTGGTATATCAACTCTCCAGCTTTATTCAGAGAGACCTTGATTGCTTTTAGCTTCCACAAAGATATCACACTGGTCCATTAAATTGATGACATTATGCTGACTGAATCCAGTGAATAAGAAGAAGTAGCAAACAAATTGGACTTATTGGTGAGACATTTGCATGCCAGAGGACAGGAAATAAATCCAACTAAAATTCAGGGAACTTCTACCTCAGTAAAATTTCTAGGGGTCCAGTGGTGTGGGGCTTGTTGAGATATTCCTTCTAAGGTGAAGGATAAGTTGCTACTTTTGGTCCCTCCTACAACCAAGAAAGAGGCACAACACCTAGTGGGCCTATTTGGATTTTGGAGGCAACACATTCTTCATT

>S-A4LNU:1:1106:13720:8316

GTCTCTGAGCTGATGTTTATTCCAGGGAACCCAAAACATCATTGTGGCCCTCCAGTTAAAGTAGGGGCTTATGGAGATCAGGTAATTAATGAAGTTGCAGCTCAGGTCTGACTTACAGTGGGTCCAGTGGGTCCAGTGGGTCCCCAGACTCATCCTGTGGTCATTTCCCCAGTGCCAGAATGGTAATTGGCATAGACATACTTAGCAGCTGGTAGAACCCCTACATTAGCTCCCTGACTGTTAGGGTGAGAGTTATTGTGGTGGGAAAGGCCAAATGGAAGCAGAGCTGTCTCTACCTAGAAAAATGATAAATCAAAAACAATATTGCATCCCTGGAGGGATTGCAGAGATTAGTGCTACCATCAAGGACTTGAAAGATGCAGGGGTGGTGATTCCTACCACATCTGCGTTCAACTCTCCCATTTGGCTGCACACCAGGT

>S-A4LNU:1:1108:5433:14444

ATTCACTCAAGGGGAGGCTGGTGTTGTGGGGCTACATGGAGATTTCCCTTCTAAGGCAGAGAACGCTTTGATGCATCTGGCCCCACACCTGCAAGTGAAAAGGAGACACAATGCCTGATAGGCCTCTATGGAGTTTGGAGGCAGTATATTTCTCATTTGGGTGCATTAGTCCAACTCATTTACTGGGTGAACCAAAAAGCTGCTAATTTGAGGGGCACAGAACAAGAGAAGTCTCTGCAACAGGTCTTGG

>S-A4LNU:1:1108:19302:8263

GTCGTAGCTGGTATTGATGACTACCTTCTTTTACTACCCATTCTGTATTCCCTTTGCCTTCAGCAAGCACCTCAGCAGGTCATGGTTTTTTTCCTGGTGGAGTGACCCAAACCTTCATTCCTGAGGGGTCTGGGCCATTTGTAGTCCTGCCTGGATTGGGTTGTTGCAGTTACCCATTGACCTTAATCATAGTGCATGGTAATACCAAGGGATGCCCTAATGGATCTCCTGTATTCCATGCATACTCTTCCTTATCTTCGTTGTGTGATAATATAC

>S-A4LNU:1:1109:26411:13916

GATGGCCTCATGGAGAGTTCCCTATGATCAGTTGACAGAGGAACAGAAGACTAGGGCCTGGTTCACAGATGGTTCTGCATGATATGCAGGCACCACCTGAAAGTGGAGAGCTGCAGCGCTACACCGCCTTTCTAGGAAATCCTCTAAAGACAGCTGTGAAGGGAAATCTTCCCAGTGAGCAGAACTTGAGCAGTGCACCTGGTTGTGCACTTTGCACAGAAGGAGAAATGGCCAGATGTGCGATTATATAATGGTTCATGGGCTGTAGGGAATGGTTTGGCTGGATGGTCAGGGACTTGGAAGAAGCATGATTGGAAAATTGGTGACAAAGAAATTTGGGGAAGAGATATATGGATGGACATCCTCAGTGGTCAAAAACTGTGAAGATATTTGTATCC

>S-A4LNU:1:1113:25773:13002

GAGCATACATGGTCTTCTAGAGAACTTTGCCCCAGCCCTGCAAAGTATTGTCATCTAGTTGGCGTTGTAATTGTGACTTCAAAAGGCCATTACACCGTTCTATCAATCCAGCTGCTTCAGGATGATGGGGAACATGGTAAGACCAGTGAATTCCATGAGCATGAACCTACTGCTGCACTTCTTTAGCTGTAAAGTGAGTGCCTTGGTCAGAGGCGATGCTGTGTGGAATACTATGATGGTGGATAAGG

>S-A4LNU:1:2110:22904:12538

CACCAAGGCTGACCTGGCTATGGCCACTGCTGAGTGCCCAATTTGCCAGCAGCAGAGACCAACACTGAGTCCTCAATATGGCACCATTCCTCGGGGTGATCATCAAGCTACCTGGTGGCAGGTTGATTATATTGGACCTCTTCCATCATGGAAAGGGCAGAGGTTTGTCCTCACTGGAATAGGCACTTACTCCAGATATGGGTTTACCTATTCTGCAT

>S-A4LNU:1:2104:20548:4355

CAACCAGGTGCACTGCTCAAGTTCTGCTCACTGGGAAGATTTCCCTTCACAGCTGTCTTTAGAGGATTTCCTAGAAAGGCGGTGTAGCGCTGCAGCTCTCCACTTTCAGGTGGTGCCTGCATATCATGCAGAACCATCTGTGAACCAGGCCCTAGTCTTCTGTTCCTCTGTCAACTGATCATAGGGAACTCTCCATGAGGCCATCAGTGCAACCTGGGGGAGAGGAGCCAGGGTGGCAGGAGTGGAGACAACGGGCACTTCCGCCACTTCCTCATGTAACTTACTTGTGCCTTCAGGACCTGCTCAAGCCTGGTAACATATACCACTTCCATTTATGATGGAATGCTGCTGTGCAT

>S-A4LNU:1:2114:28971:10847

ACTGCTGAGTGCCCAATTTGCCAGCAGCAGAGACCGACATTGAGCCCTTGATATGGCACAATTTCTCAGGGTGATCAGCCAGCTACCTGGTGGCAGGTTGATTATATTGGACCTCTTCCATCATGGAAAGGGCAGAGGTTTGTCGTTACTGGAATAGACACTTACTCCAGATATGGGTTTGCCTGTCCTGTATGAAATGCTTCTGCCAAGACTACCATCTGTGGACTTACGGAATGCCTTATCCACCATC

>S-A4LNU:1:1102:11143:8448

GTGGTGTCCATAGAATGGATCATCCTATCCACTTGATTATTAAAACCCTCCTCTGCTGAGGTCACCCGTTGGTGAGCACTCACATGGGATACCAATATCTTCACAGTTTTTGACCACTCAGAGAGGTCCATCCACACACCTCTTCCCCAAATTTCTGTCACCAATTTTCCAACCATGCTTCTTCCAAGTCCCTGACCATCCAGCCAAACCACTGGCTACAGGCTGTGAATCAGAATATAATAGCATATCTGGCCATTTCTCCTTCCATGCGAAGTGCACAGCCATGTGCACTGCTTGAAGTTCTGCCCACTAGAAAGATTTCCCTTCATCACTGTCCTAGAAAGAGGCTGTAGTGCTGCAGCTGTCCACTTTCGGGTGGTGCCTGCACATCTTGCAGAGCCATCTGTGAACCAGGCCCTAGTCTTCTCTTCCTCTGTCAATTGATCATAGAG

>S-A4LNU:1:1107:13012:9571

GCACTCGTATGGGATACAAATATCTTCACAGTTTTTGACCACTGAGGATGTCCATCCATATATCTCTTCCCCAAATTTCTTTGTCACCAATTTTCCAATCATGCTTCTTCCAAGTCCCTGACCATCCAGCCAAACCATTCCCTACAGCCCATGAACCATTATATAATCGCACATCTGGCCATTTCTCCTTCTGTGCAAAGTGCACAACCAGGTGCACTGCTCAAGTTCTGCTCACTGGGAAGATTTCCCTTCACAGCTGTCTTTAGAGGATTTCCTAGAAAGGCGGTGTAGCGCTGCAGCTCTCCACTTTCAGGTGGTGCCTGCATATCATGCAGAAC

>S-A4LNU:1:1106:15913:9320

GTACCAGGAGACGCGTTAATTTGCTCAAGCAATGAAACCACTTCTGGTACAGCAGCTGCAATTGGAGTCACCACGTGGTTACTGTCTTCTGTGCAGGCCAAATGGGAGAGTTGAACGCAGATGTGGTAGGAATCACCACCCCTGCATCTTTCAAGTCCTTGATGGTAGCACTAATCTCTGCAATCCCTCCAGGGATGCAATATTGTTTTTGATTTATCATTTTTCTAGGTAGAGACAGCTCTGCTTCCATTTGGCCTTTCCCACCACAATAACTCTCACCCTAACAG

>S-A4LNU:1:1114:21164:2517

CCTTAATCACATGGCATGGTAATATTAAGAGATGCCCTAATGGATCTCCTGTGTTCCATGCATACTCTTCCTTACCTCCATTATGGAATAGCAGACTGATTTCATCTTGACAGTCCAGGTCAATCAGCCCAGCCAACACTGTAAATCCCTTCTTATCCTGTTGACTTTAGGATAGGAGCAGCCCAAAGTGTCCAGGTG

>S-A4LNU:1:1112:11072:14753

ATCCATATATCTCTTCCCCAAATTTCTTTGTCACCAATTTTCCAATCATGCTTCTTCCAAGTCCCTGACCATCCAGCCAAACCATTCCCTACAGCCCATGAACCATTATATAATCGCACATCTGGCCATTTCTCCTTCTGTGCAAAGTGCACAACCAGGTGCACTGCTCAAGTTCTGCTCACTGGGAAGATTTCCCTTCACAGCTGTCTTTAGAGGATTTCCTAGAAAGGCGGTGTAGCGCTGCAGCTCTCCACTTTCAGG

>S-A4LNU:1:1110:10464:25355

TCATGAATGTGACGTTGTTTTTGACGTACTATTTTTCTAAGTAGAGGCAGCTCTAATGGCTTCCATTTGGCCTTTCCTACCACGATAGCCCTCACCTTACCAGTCAGGAAGCCAATGTGGGGGTTCTGCCAGCTGCTAAGTATGTCTATGCCAATTATGCATTCTGCCACTGGAGAAATGACCAGAGGATGAGTCCAGGGACCAACTGGATGTACTGTACATCAGACCTGAGCTAAAACTCCATTAATTACCTGACTTCCATAAACCCCTACTCTAACTGGAGAACCACAATGATGCTTTGGGTCCCTTGGAATCAACATCAGCTCTGAGCCAGTGTCCAGTAGCACCCCCAAGTGTCTGACTATTTCCCTTTCCCCAGTGCACTTTAGCCTGCTAAAAGGCCAGAGGTCTATGAAGGGAAGGATGGGAGAGAGATTCAC

>S-A4LNU:1:1113:25652:10479

CCCAAGTAGCTGGGATTATGGGCACCTCCCACCACACCCGGCTAATTTTTTGTATTTTTAGTAGAGATGGGGTTTCACCATGTTGGCCAGGATGGTCTTGATCTCTTGACCTCTTGTGGAGGAGAAAAGCTATCAAGGTCTCTCCAAATAAGATTATGACACAAAGCCTGAGAGTTGATATACCCCTGAGGTAGGACAGTAAAGGTATATTGCTGGCCTTGTCAGCCGAAGGGAAATTGCTTCTAGTAGGCCTTATGGACAGGAATGGAGAAAAAGGCATTTTCCAAGTAAATGGCTGCACACCAGGTACCAGGAGACGCGTTAATTTGCTCAAGCAATGAAACCACTTCTGGTACAGCAGCTGCAATTGGAGTCACCACGTGGTTACTGTCTTCTGTGCAGGCCAAATGGGAGAGTTGAACGCAGATGTGGTAGGAATCACCACCCCTGCAT

>S-A4LNU:1:2111:13229:13285

GTGTAATGGCCTTTTGAAGTCACAATTACAACGCCAACTAGATGACAATACTTTGCAGGGCTGGGGCAAAGTTCTCTAGAAGACCATGTATGCTCTGAATCAGTGTTCAATATATGGTACTATTTCTCCCATAGCAAGGATTCACGGGTCCAGGAATCAAGGGGTGGAAATGGAAGTGACACCACTCACCATCACCGCTAGTAATCCACTAGCAAATGTTTGCTTCCTGTTTCCCTGACATTATGTTCTCCTGGCCTAGAGGTCTTAGTTCCAGAGGGAGGAATGCTGCCACTAGGAAACACAACAAAGTTTCCATTAAACTGGAAGTTAAGATTTCCACCTGGACACTTTGGGCTGCTCCTATCCTAAAGTCAACAGGATAAGAAGGGATTTACAGTGTTGGCTGGGCTGATTGACCTGGACTGTCAAGATGAAATCAGTCTGCTATTCCATAATGGAGGTAAGGAAGAGTATGCATGGAACACAGG

>S-A4LNU:1:2105:16425:18793

GTTTACTGCAGTACTATTCACAATAGCCAAGATTTGGAAGGAACCTAAGTGTCTATCAACAGATGAGTGGATAAAGAAAATGTGATACATATACACAATGGAGTACCATTCAGCCATAACACAGAATGAGATCCTGTCATTTGCAACAACATGGGTGGAACTAGAGGTAGTTATGTGATGTAAAATAAGCCAGGCACAGAAAGACCAACTTCTCATGTTCTCACTTATTTGTGGGAGCTAAAAAAAATTAAAACAGTGAAACTCATGGAGATAGAGAGTAGAAGGATGGTTACCAGAGGCTGGGAAGGGTACTGGGGTTGGAGTCGGGGAGTGGGGATGGTTAACTGGTACAAAAATATAGTTAGATAGGATGAATAAGATCTAGTATTTGATAACACGACAGGATGACTAGAGTCAACAATAATTTATTGTATGTTTTAAAATAACTAAAAGAATAGAAT

>S-A4LNU:1:2109:18154:11273

CCCTCCCACAACACGTGGGAATTATGGGAGCTACAATTCAAGATGAGATTTGGGTGGGGACATTGCCAAAGCATATCAGTATATATCAAAAAGAAAGGAGATTAATGTATCAAAGAGATATCTGCACCTCCATGTTTACCCAAGTACCATTCACAATAGCTAAAATATGGAACCAACCTGAGTGCCCATTAACAAATGAATGAATAAAGAAAATGTGGTATATACACACAATGGAATATTTAATTCAACCATAAAAAAGAATGAAATCCTGTCATGTGCAGCAACATGGATGGAACCACAGGCAAGGTTAAGTGAAATAAGCCCAGCAGAGAATGACAAATGTCACATGTTCTCACTCTTACATGAGAGCTAAAAATGTAGATC

>S-A4LNU:1:1110:23150:7993

GCCTGGCATATTTCACTTAGTATAATGACATCCAGTCTCATCCATGTCATTGCAAATGACAGGATCTCATTCTTTTTTATGGCTGAATAGTATTCCATTATGTATATGTACCACATTTTCTTTATTCATTCATCCGTTGATGGACACTTAGGTTACTTCCAAATTTGTGTTATTGTGAATGGTGCTACAACAAACATGGGAGTGCAGATATCTCTTTTATATACTGATTTCCTTTCTTTTGGATATATACCAGCAGTGAGATTAGCAATAAAGTATTTTTAATTAAGATATGTACATTGGCATATATATATAAATATAAAGTATATATACAC

>S-A4LNU:1:2101:11587:11252

GTTCTTTTACTTATTCTATAAGGTCTAAACATAAAAAGGTTTTAGTACTGGTACCAAAAGTTAAACTTGAAGAGAATACCGAGTACATAGCTTTTTTTTTACTATAGACTACTTAGGTACTTAGTACTTACCTCTTAGAACAAGCAGTCATATTCATTTTGATTGTAGGCCACGGCTCGTAACCTTCCACTACAAAATCTCCAACCTCAAATCCATCAATTCCACCAACTGTCGCGTCTTCTCTTGTCTTTTTAATTCGGAGTTTTGGAAACTGCCTCGGCTCCCGATCAATCTGTACCTTCAGTGCCTCCACATGATCCAAATACACGTGGGCGTCACCCATCGTATGGATGAATTCACCAGCATCTAGGTCGGTAACGTGCGCAATCATATGTGTGAGAAGAGCGTAGGAGGCAATGTTAAATGGCACCCCCAGACCCATGTCGCACGAGCGTTGGTAGAGATGACATGACAGCTCACCACGG

>S-A4LNU:1:1108:9314:13541

ATAGTAAGATATCCAAATATCCCATCGCAGTTGGATTTAGAGTATGATATTATTTAGCTTCCAGCTAATGAGATGGTATCAACCGTGTTCATTAATATCTGTATTTTTCAAATATCCTTTTTTAAGCAATTCACTTTTGATATAGGGAAATAAGTCACATGTATACGGTATTTCAATAAGATCTATACTCTCTCTTTTACATACATCTCGCTTTTTTTGATCGCGCATCTGTTGACTGTCTAAGTCTATCCTGCCTCGCTTATGGAACATTGGATTCTCAGAAAAATGTTGAGGTCCCTGGAATTCATACGCCAATTTTAATTCTTTATTATACCCGTCTAATTGCATCCCATTCAAGAATAACGGTTTACAAG

>S-A4LNU:1:2106:13882:25479

CAGTACTTTCGGACAATAGAACTCCTTTAACATCTCCATATGCTTCCCAGTCAATGGATGCTCCCACATAAGCGTATTCATTGCTGGAGCAACTATCATCTCCTTCGTCCGGTCCCAAGCTCTGATAATTCCTCCCAATAACCCTTCCACATGACCATTGACCATCTTTGCCATAAAATTTGCGCTGAGTGGTGCAATCACAAGTAGATCTGCCCATTTCCTGAGCTCGATATGAAGAACCGGATCTCCAATTGTTCCCCACTGATTCCACTCATCCTCATCCGTCCAA

>S-A4LNU:1:1113:10929:20186

GGGAGGGATTGATAGGTGGAAACTTCAGTGGGAGAGTAAGTAGGAATGACTGATGAGAAAGAGAAAAACCGGCCGTGAGTGAAAGAAGTAGGAATACTGGCTGCTTCTTTTGCTGTCTTATCAGCATAATTGTTGCCTTGAGCAATGGGGTCTGAGGCCATTTGATGGCCTTTGCAGTGAAGGACTCCAGCTTCCTTTGGAAGTAAAGCAGGCTTGTGAAGAGTTTTCGTTAAAGAGGCATTAATGATGGAGGACCCTTGCGTAGTGAGAAAACCTCTTTCAGCCCATATAACAGCATGGTGGTGCAGGATATGGAAGGCATATATAAAGTCAGTATAAATATTGACATGCAGTCATTTTGCAAGAGTGAGGGCTTCAGTTAAGTCTAGGAGTTTGGTTTGCTGAGAGGTAGTGGAG

>S-A4LNU:1:1101:14085:3628

ATCCATTACTCTCTGGAACGTTCCTGGTGCATTCTTCAATCCAAAAGGCATAACATTATATTCAAATAACCCTTGTGAACATATAAACACTGTTTTCTCTTTATCCTCTTCGGCTACCTCTATCTGATGATATCCTGCAGCCAAATCTAAACTCGTAAACCATTTTGCATCCTTATAC

>S-A4LNU:1:1104:25363:18971

GTATGAAGCCTGCCTAGTTGCTAAAGGATTCTTTTAATAACCTGAATTGGACTATAAAGAGATTTATTCTCCAGTTATCAGGATGGACTCCCTTCACTTACTACTTGCCCTCTCTATATATTCCAAGTGGACTCCTCAACAACTCGATATTAAAACTGCCTTTCTCTATGGCAACTTAAAGGAAGATATCTATATGCATCTACCTCCTGGCAGCAGAGGTGATGGTATGATGGTTACTAAACTAAAAATGTATATCTATAGACTTAAGCAATTACCAAGAGAATAGTATGCACAGCTCATTATCTAC

>S-A4LNU:1:1103:12506:5820

GTCTTTTGTCTATTTGACCTAGAAGTTTTCTCCTTACATAAAGTAAGTAGGAATGCAGTAGGCTTCCTATCAATTTCACTTCTAGGAACACCACGGTTAATTAGTCAATGCCAGAGCTTTACATGAGTCAGACTACTCTGATCGCTGCTTTGTCTCTGCTGCCCATTATGGTAGCTCACCTTGCCTGTCACAGTTGAGTGCTGCCACTTGGCCTCTGCCACCTCGGGATCCAATTATTCCCACTGTATTTAAATTTTGTAGTTGAGTGACTGTGGTTCCCACTGTTAGATCTAATATACAGAGAAGAGCAATTACACGGTTCTTCAAAGATGCAGGTGCTGCCCTCACAAATCTATTTTGCAAGGCATTGGTCAAGGGTATATCTTCTGGATACTCCCAGCTGGGATGAGTAGGTCTAATGTGACT

>S-A4LNU:1:1104:21289:25308

AGGTGGCAGAGGCCAAGTGGCAGCACTCAACTGTGACAGGCAAGGTGAGCTACCATAATGGGCAGCAGAGACAAAGCAGCGATCAGAGTAGTCTGACTCATGTAAAGCTCTGGCATTGACTAATTAACCGTGGTGTTCCTAGAAGTGAAATTGATAGGAAGCCTACTGCATTCCTACTTACTTTATGTAAGGAGAAAACTTCTAGGTCAAATAGACAAAAGACTAATTTGAATTATAAAAACAGAGAATCGTGGCCCCTCAATCAATTTCCAGACTTGAGTCAGTTTACAGGCCCAGAACCCCTTGAATGAAAGGAATGCCGGATCCTCTTAAGGAAAGATCCTACTATATTACTGATAATTTATGCAGTGAATCTTTCTCCCATCCTTCCCCAAGGACACCTCTGGCTTTTTACCAGGGTAACTGTGC

>S-A4LNU:1:2112:14158:11077

ATGTAGAGGAAGGGATGCAAAGGCTTAGGGAGATTGGGATGGTGCATTAGTCACATTAGACCTACTCATCCCAGCTGGGAGTATCCAGAAGATATACCCTTGACCAATGCCTTGCAAAATAGATTTGTGAGGGCAGCACCTGCATCTTTGAAGAACCGTGTAATTGCTCTTCTCTGTATATTAGATCTAACAGTGGGAACCACAGTCACTCAACTACAAAATTTAAATACAGTGGGAATAATTGGATCCCGAGGTGGCAGAGGCCAAGTGGCAGCACTCAACTGTGACAGGCAAGGTGAGCTACCATAATGGGCAGCAGAGACAAAGCAGCGATCAGAGTAGTCT
